# Diagnostic Performance of Clinicopathological Analytes in *Otostrongylus circumlitis-*Infected Rehabilitating Juvenile Northern Elephant Seals (*Mirounga angustirostris*)

**DOI:** 10.3389/fvets.2019.00134

**Published:** 2019-04-25

**Authors:** Julie D. Sheldon, Jorge A. Hernandez, Shawn P. Johnson, Cara Field, Sarrah Kaye, Nicole I. Stacy

**Affiliations:** ^1^Illinois Zoological and Aquatic Animal Residency Program, Urbana, IL, United States; ^2^Department of Large Animal Clinical Sciences, University of Florida College of Veterinary Medicine, Gainesville, FL, United States; ^3^The Marine Mammal Center, Sausalito, CA, United States; ^4^Staten Island Zoo, Staten Island, NY, United States; ^5^Department of Comparative, Diagnostic, and Population Medicine, University of Florida College of Veterinary Medicine, Gainesville, FL, United States

**Keywords:** clinical pathology, DIC, *Mirounga angustirostris*, Northern elephant seal, *Otostrongylus circumlitis*, ROC analysis

## Abstract

The nematode lungworm, *Otostrongylus circumlitis* (OC), is a significant cause of northern elephant seal (NES; *Mirounga angustirostris*) mortality at The Marine Mammal Center (TMMC, Sausalito, CA). The current lack of specific antemortem diagnostic tests for pre-patent OC infection in NES makes diagnosis, proper treatment, and assessment of efficacy of medications challenging. Severe inflammation and disseminated intravascular coagulation (DIC) develop rapidly and are difficult to treat once clinical signs develop. Certain blood inflammatory and hemostasis biomarkers for early diagnosis have recently been investigated. The objective of this study was to investigate the diagnostic performance of complete blood count, serum chemistry, acute phase proteins, protein electrophoresis, and coagulation parameters for diagnosis of OC clinical infection in NES. Samples from NES with OC infection confirmed by gross pathology with blood collected antemortem during clinical disease (*n* = 9) and NES initially admitted for malnutrition and sampled shortly before release after successful rehabilitation (*n* = 20) were included in the study. Using Receiver operator characteristic (ROC) curve analysis, the diagnostic performances (area under the curve [AUC]) of albumin (0.994), albumin:globulin ratio (0.983), serum amyloid A (0.972), activated partial thromboplastin time (0.936), total bilirubin (0.975), and gamma-glutamyl transferase (0.939) were high (AUC > 0.9). These results confirm systemic inflammation and DIC, and support previously reported clinical and gross pathological findings in NES infected with OC. In addition to AUC values, this study produced cut-off points, sensitivity, specificity, confidence intervals, and predictive values for analytes with high diagnostic performance. This data will be useful in the diagnosis and clinical management of OC-infected NES and will aid in assessment of treatment efficacy.

## Introduction

The Northern elephant seal (*Mirounga angustirostris;* NES) is a phocid species inhabiting the California coast. Despite an increasing population at about 200,000 animals, this species was almost driven to extinction in the 19th century due to commercial hunting. As a result, this population underwent a bottleneck effect, decreasing the genetic diversity (1). About 150 juveniles are admitted each year to the Marine Mammal Center (TMMC) for rehabilitation due to malnutrition, trauma, human interaction, and infectious diseases (2).

Infections with the nematode lungworm, *Otostrongylus circumlitits* (OC), are responsible for 12% of NES strandings and 37% of NES mortality at TMMC (2). OC has an indirect lifecycle and a 3rd stage larvae in a fish intermediate host. Seals consume the fish and the ingested larvae migrate from the gastrointestinal tract to the liver, heart, and lungs. Once clinical signs become apparent, severe inflammation and disseminated intravascular coagulation (DIC) develop rapidly and are difficult to resolve therapeutically. Clinicopathological changes usually occur after or in concert with clinical signs (1). No antemortem diagnostic test currently exists as mortality occurs prior to ova being shed in the gastrointestinal tract, and no other pathogen identification tests have been successfully developed (1, 3).

Recent studies have evaluated the use of blood inflammatory markers, such as acute phase proteins, and hemostatic parameters, in guidance to early therapy (3, 4). However, definitive clinical diagnosis cannot be made until nematodes are grossly identified on necropsy (1, 2).

The objective of this study was to investigate the diagnostic performance of complete blood count, plasma chemistry, acute phase proteins, protein electrophoresis, and coagulation parameters for diagnosis of OC clinical infection in NES.

## Materials and Methods

The animals included in this study were retrospectively selected based on antemortem blood work and archived serum sample availability. Samples were collected in 2014–2015. The healthy group included blood samples obtained from 20 juvenile apparently healthy NES initially admitted to TMMC for malnutrition only and sampled within 3 days before release after successful rehabilitation. Animals were determined as healthy for release based on lack of historical abnormalities except for malnutrition upon admission and normal physical examination, in addition to absence of any derangements based on complete blood count (CBC) and serum chemistry. The OC-infected group included blood samples obtained from nine juvenile NES with OC infection as cause of death confirmed by gross pathology and available archived blood from antemortem collection during clinical disease.

Whole blood from all NES was previously collected antemortem under manual restraint from the extradural intervertebral sinus using a 20 Ga 1.5” needle on a Vacutainer set (Becton Dickinson, Franklin Lakes, NJ, USA) into EDTA, serum separator, and citrate tubes. The following tests were performed on all 29 samples: CBC (total leukocyte count, red blood cell count, hemoglobin concentration, hematocrit, platelet concentration, mean cell volume, mean cell hemoglobin, mean corpuscular hemoglobin concentration, red cell distribution width, mean platelet volume, and absolute differential counts for neutrophils, eosinophils, lymphocytes, monocytes, and basophils) and plasma biochemistry (gamma glutamyl transferase [GGT], alanine aminotransferase [ALT], aspartate aminotransferase [AST], alkaline phosphatase, creatine kinase, total bilirubin, glucose, phosphorus, blood urea nitrogen [BUN], creatinine, BUN:creatinine, calcium, sodium, potassium, chloride, total protein, albumin, globulin, and albumin:globulin [A:G] ratio) at TMMC, acute phase proteins serum amyloid A (SAA) and C-reactive protein (CRP) measured via immunoturbidimetric assays and protein electrophoresis (prealbumin, albumin, alpha1, alpha2, beta, and gamma globulins) at the University of Miami Avian and Wildlife Laboratory, and coagulation parameters (D-dimer, activated partial thromboplastin time [APTT], prothrombin time [PT], fibrinogen, antithrombin) at the Cornell University College of Veterinary Medicine via methods described in previous studies (3, 4).

Receiver operator characteristic (ROC) curve analysis was used to assess and compare diagnostic performance of all of the analytes listed above. Cut-off points, sensitivity (%), specificity (%), area under the curve (AUC), and 95% confidence intervals (CI) were also calculated for each analyte (MedCalc Statistical Software version 14.10.2; bvba, Ostend, Belgium; http://www.medcalc.org; 2014). Using ROC analysis, a perfect test (100% sensitivity and 0% false positives) has an AUC = 1, highly performing tests: 0.9 < AUC <1, moderately performing tests: 0.7 < AUC ≤ 0.9, low performing tests: 0.5 < AUC ≤ 0.7, and non-informative tests: AUC = 0.5 (5).

## Results

**Figure 1:**
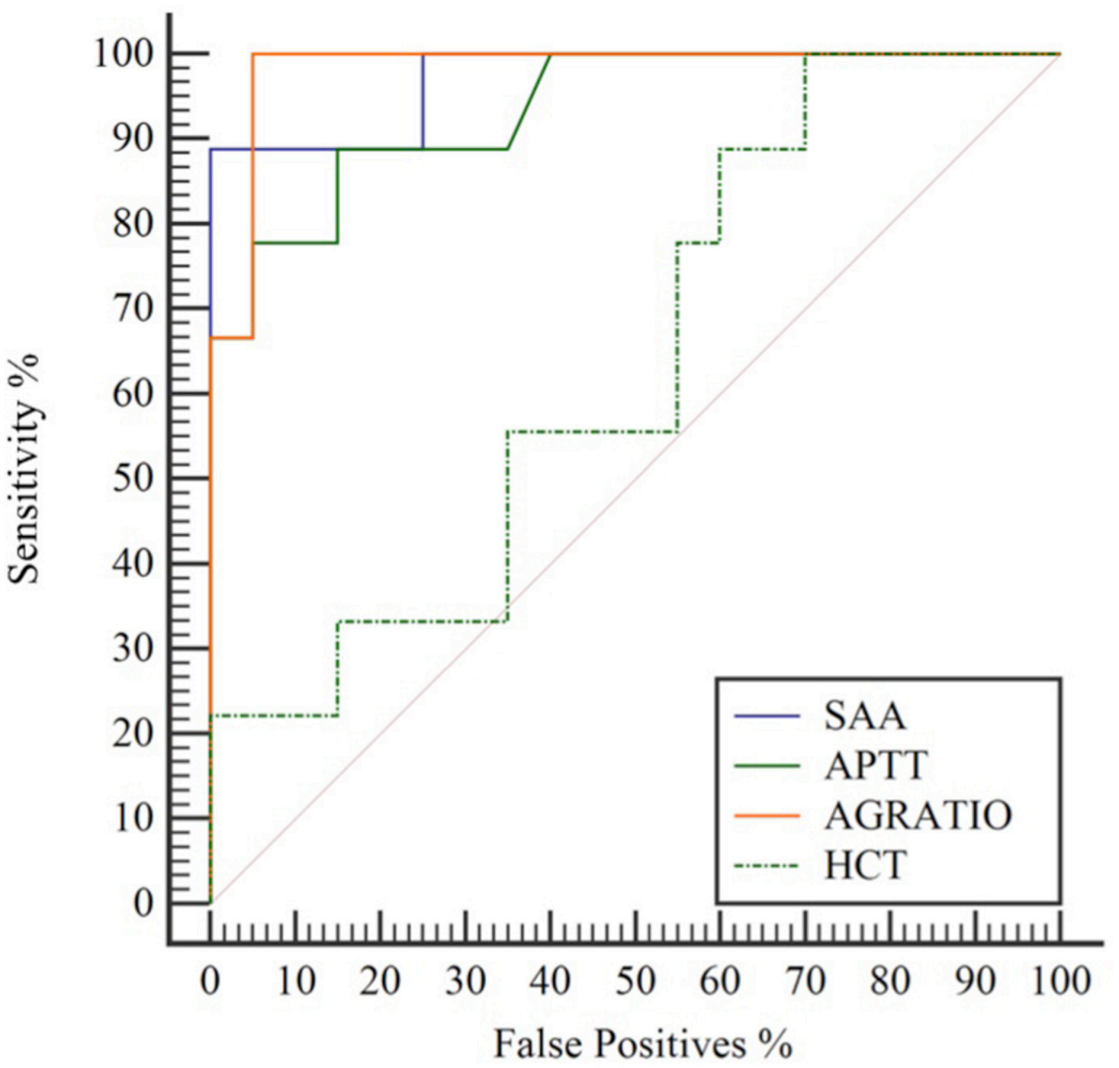
Comparison of receiver operating characteristic (ROC) curves depicting highly accurate serum amyloid A (SAA; blue solid line), activated prothrombin time (APTT; green solid line), and albumin:globulin ratio (A:G ratio; orange solid line) in contrast to less accurate hematocrit (HCT; green dashed line) for diagnosis of *Otostrongylus circumlitis* clinical infections in Northern elephant seals (*Mirounga angustirostris*).

Several tests showed high diagnostic performance (AUC > 0.9) in the diagnosis of OC infection in NES. Fourteen analytes had an AUC > 0.9, and 14 had an AUC < 0.9 and >0.7. All 41 analytes evaluated had an AUC of > 0.52. A:G ratio (decreased), serum amyloid A (increased), and activated partial thromboplastin time (increased) were among the best performing analytes. Hematocrit, beta globulins, and BUN were among more poorly scoring analytes. Figure 1 shows the ROC curve of 3 highly accurate analytes (SAA, APTT, and A:G ratio) compared to a less accurate analyte, HCT. Analyte cut-off points, sensitivity, specificity, and AUC in order of decreasing performance are listed in Table 1.

**Table 1:** Cut-off points, sensitivity, specificity, area under the curve (AUC), and 95% confidence interval (CI) from highest to lowest AUC in the diagnosis of *Otostrongylus circumlitis* clinical infection in Northern elephant seals (*Mirounga angustirostris*).

**Analyte**	**Cut-off point**	**Sensitivity %**	**Specificity %**	**AUC**	**95% CI**
Calcium (mg/dl)	≤9.3	100	100	1	0.881, 1.000
Albumin (g/dl)	≤2.6	100	95	0.994	0.870, 1.000
Albumin:Globulin ratio	≤0.69	100	95	0.983	0.851, 1.000
Total bilirubin (mg/dl)	>0.7	88.9	95	0.975	0.837, 1.000
Serum amyloid A (mg/l)	>20.08	88.9	100	0.972	0.833, 1.000
Potassium (mmol/l)	≤4.5	88.9	95	0.967	0.824, 0.999
Gamma-glutamyl transferase (U/l)	>83	100	85	0.939	0.783, 0.994
Sodium (mmol/l)	≤144.5	88.9	90	0.939	0.783, 0.994
Activated partial thromboplastin time (sec)	>27	88.9	85	0.936	0.779, 0.993
Neutrophils (cells × 10^3^/μl)	>12.93	100	75	0.93	0.85, 1.00
White blood cell count (cells × 10^3^/μl)	>27.4	77.8	95	0.917	0.753, 0.986
Mean platelet volume (fl)	>9.2	88.9	95	0.914	0.749, 0.985
Band neutrophils (cellsx10^3^/μl)	>1.33	88.9	100	0.91	0.76, 1.00
C-reactive protein (mg/l)	>9.04	88.9	100	0.906	0.738, 0.982
Prothrombin time (s)	>17.4	88.9	90	0.883	0.710, 0.972
Aspartate aminotransferase (U/l)	>66	88.9	90	0.878	0.703, 0.969
Alpha 2 globulins (g/dl)	>1.42	66.7	95	0.858	0.679, 0.959
Alanine aminotransferase (U/l)	>45	77.8	95	0.858	0.679, 0.959
Mean cell volume (fl)	≤179	88.9	75	0.853	0.672, 0.956
Fibrinogen (mg/dl)	≤98	77.8	100	0.839	0.656, 0.948
Alpha1 globulins (g/dl)	≤0.3	88.9	80	0.833	0.649, 0.945
Platelet count (cells × 10^3^/μl)	≤309	77.8	95	0.831	0.646, 0.943
Creatine kinase (U/l)	≤264	77.8	95	0.825	0.639, 0.940
Gamma globulins (g/dl)	>0.84	77.8	85	0.8	0.611, 0.924
Total protein (g/dl)	≤6	77.8	75	0.772	0.580, 0.906
Reticulocyte distribution width (%)	>5.7	100	70	0.767	0.573, 0.903
MCHC (g/dl)	>37.4	100	50	0.719	0.523, 0.869
Antithrombin III (%)	≤81	66.7	90	0.708	0.511, 0.861
Blood Urea Nitrogen:Creatinine ratio	≤100	88.9	50	0.681	0.482, 0.840
Glucose (mg/dl)	≤138	55.6	100	0.669	0.471, 0.832
Phosphorus (mg/dl)	>6.8	55.6	85	0.661	0.463, 0.825
Blood Urea Nitrogen (mg/dl)	≤28	66.6	75	0.656	0.457, 0.821
Hematocrit (%)	≤58.1	100	30	0.639	0.440, 0.808
Eosinophils (cells × 10^3^/μl)	≤0.00	77.8	50	0.63	0.42, 0.85
Basophils (cells × 10^3^/μl)	≤0.00	100	20	0.6	0.38, 0.81
Beta globulins (g/dl)	>10.7	33.3	95	0.572	0.376, 0.753
Pre-albumin (g/dl)	>0	44.4	65	0.533	0.340, 0.720
Hemoglobin (g/dl)	>21.2	66.7	55	0.522	0.330, 0.710
RBC count (cells × 10^6^/μl)	≤3.02	33.3	45	0.522	0.330, 0.710
Lymphocytes (cells × 10^3^/μl)	>2.50	66.7	0	0.52	0.24, 0.81
Monocytes (cells × 10^3^/μl)	≥0.38	55.6	70	0.52	0.25, 0.78

*MCHC, mean corpuscular hemoglobin concentration; RBC, red blood cell*.

## Discussion

Diagnosis and treatment of OC infections in NES continue to pose challenges due to severity of clinical signs, difficulty of medically reversing DIC, and lack of diagnostic techniques to identify parasitic infection antemortem. This study offers new information about the diagnostic performance and cut-off points for a suite of clinicopathological analytes to aid in diagnosis, gauging disease severity, and monitoring the progress of clinical cases during rehabilitation. Treatment of suspected cases of OC in NES includes antibiotics, salicylic acid, ε-aminocaproic acid, and anti-inflammatory doses of corticosteroids. Traditionally, anthelminthic treatment has been avoided due to the risk of peracute death from rapid release of parasitic antigens (6). The pathophysiology of OC in NES resembles that of *Angiostrongylus vasorum* in dogs, which is helping to guide current investigations into appropriate anthelminthic treatment protocols in suspected OC cases at TMMC (7). However, current studies are investigating the use of anthelminthics as treatment in suspected cases of OC infected NES at the Marine Mammal Center.

Although several of the analytes performed well, they may not necessarily be specific to OC-infections given the complex sequelae of systemic inflammation and DIC. However, in using these readily available diagnostic tests collectively and in context of the identified cut-off points and clinical findings, blood analyte derangements can be used as efficient diagnostic and monitoring tools.

Among the best performing variables in this study are most inflammatory biomarkers, notably albumin, A:G ratio, SAA, and CRP, thus reflecting systemic inflammation, one of the major underlying pathophysiologic mechanisms in OC clinical cases. Both albumin and A:G ratio were decreased in OC-infected animals. Albumin is a negative acute-phase protein that is known to decrease in the presence of inflammation due to decreased hepatocyte production in response to interleukins 1 and 6 (8). Since globulins tend to increase in OC-infected animals, the A:G ratio will also drop with concurrently decreased albumin. The acute phase proteins SAA and CRP also performed well. Acute phase proteins are being utilized increasingly in wildlife species for early detection of inflammation since they have proven useful in identifying species-specific responses to inflammatory processes (9, 10). For example, they are used to characterize and monitor inflammation in several marine mammal species including manatees, dolphins, and harbor seals (11–14). One study found that SAA is significantly increased in pre-clinical and clinical OC-infected NES, while CRP protein only increased in clinical OC-infected NES; therefore, it is not surprising that SAA and CRP both performed well in this present study (3). The fact that acute phase proteins scored higher than any leukogram variables emphasizes the importance of monitoring early inflammatory markers outside of the leukogram.

The highest performing leukogram parameter was neutrophil concentration, followed by total white blood cell count and absolute band neutrophils. However, based on clinical experience at TMMC, NES generally do not show any overt leukogram changes until after clinical signs of OC-infection manifest, rendering these changes not clinically useful in early diagnosis as previously described in another study (3).

The clinical relevance of coagulopathies in OC-infected NES was supported by the well-performing analytes APTT and PT, both of which are typically prolonged in DIC. APTT measures the functionality of the surface-induced intrinsic and common pathways while PT measures that of the extrinsic and common pathways of the coagulation cascade (8). A previous study found that the OC-infected animals were hypocoagulable, mainly with prolonged APTT and PT (4). However, platelet concentration and antithrombin III did not perform as well in our study. Several OC-infected NES had platelet counts within the normal range and none suggested the animals were at risk of spontaneous hemorrhage based on coagulation physiology in domestic mammals (<30,000 cells/μL) (8).

Underlying hepatic impairment in OC-infected NES is demonstrated by the high diagnostic performance of bilirubin and liver enzymes. Increased total bilirubin in OC-infected NES could be due to decreased uptake and conjugation by hepatocytes resulting from anorexia and/or hypoxic liver damage, hepatitis/cholangitis from systemic inflammation, or extravascular hemolysis and consequent increased unconjugated bilirubin uptake (8). Increased liver enzyme activities have been described previously in OC-infected NES (1). GGT, AST, and ALT also performed well and support the conclusion of hepatocellular damage as a significant component of systemic disease. Since nematodes have not been found outside of the heart and pulmonary vasculature, these findings are presumably associated with systemic inflammation (1).

Several other analytes performed very well; although, these are generally non-specific for OC infection, are most consistent with sequelae of systemic inflammation, or reflect downstream effects of more clinically significant clinicopathological derangements. For example, calcium concentrations in OC-infected NES ranged from 7.9–9.3 mg/dl and all healthy NES had calcium concentrations > 9.3 mg/dl. The degree of hypocalcemia in the study animals was not severe enough to cause clinical signs specific to hypocalcemia such as tremors/seizures. Hypocalcemia in these animals was likely due to decreased albumin since calcium is bound to albumin in circulation. Other possible causes of hypocalcemia include hypoparathyroidism, inadequate mobilization from bone or absorption in the intestine, or excess urinary excretion (8). Potassium and sodium both decreased in OC-infected NES and performed highly with AUCs of 0.967 and 0.939, respectively. While the magnitude of the changes were not clinically significant on their own they may reflect intra- and extracellular shifts due to loss in vomit, diarrhea, or renal excretion (8). The remaining analytes are less useful in diagnosing and monitoring disease progress of OC-infected NES but derangements thereof may be effects of severe systemic disease.

The number of highly performing analytes in this study was remarkable and highlights the severity of systemic effects of OC on NES. Results of this study support the presence of systemic inflammation and DIC in affected NES, and the previously reported pathological findings in NES infected with OC. It is presumed that NES is a relatively new host to OC and is unable to survive infection long enough for the nematodes to undergo a full lifecycle (15, 16). The data presented herein will be useful in clinical management and will aid in assessment of treatment efficacy in OC-infected NES. Acute phase proteins and coagulation parameters demonstrated superior diagnostic performance over CBC data, which emphasizes the importance of including SAA and CRP as part of the complete health evaluation in stranded NES and for early diagnosis of OC infection. Based on the results of this study and previous reports, in addition to clinical experience and anecdotal information, authors recommend that albumin, AG ratio, APTT, total bilirubin, and GGT are the most useful clinicopathological markers for the diagnosis of OC- NES. Until a definitive antemortem diagnostic test is developed, analyzing appropriate clinicopathological findings in context of all relevant clinical information is considered crucial for rapid implementation of therapy, monitoring during treatment, and assessing efficacy of various treatment modalities.

## Ethics Statement

This study was carried out in accordance with the Stranding Agreement between the National Oceanic and Atmospheric Administration's National Marine Fisheries Service West Coast Region and the Marine Mammal Center approved for November 2013 through December 2015.

## Author Contributions

JS was responsible for primarily writing this manuscript, initial animal, and sample selection, and result interpretation. JH performed statistical analysis. SJ is veterinary director for the animals used in this study and oversaw veterinary care for them. CF is a clinical veterinarian for these animals, provided instrumental information regarding current treatment and monitoring of patients with this disease, and helped perform clinical exams and sample collections for this study. SK was responsible for performing all coagulation parameter tests and analysis. NS oversaw all aspects of the project and provided expertise in data analysis and interpretation. All coauthors played a significant role in the editing/reviewing of this paper.

### Conflict of Interest Statement

The authors declare that the research was conducted in the absence of any commercial or financial relationships that could be construed as a potential conflict of interest. The reviewer NL declared a past supervisory role with one of the author JS to the handling editor.
